# Emerging Principles of Selective ER Autophagy

**DOI:** 10.1016/j.jmb.2019.05.012

**Published:** 2020-01-03

**Authors:** Simon Wilkinson

**Affiliations:** Edinburgh Cancer Research UK Centre, MRC Institute of Genetics and Molecular Medicine, University of Edinburgh, EH4 2XR, United Kingdom

**Keywords:** TEX264, SEC62, FAM134B, CCPG1, RTN3L, ER, endoplasmic reticulum, rER, rough ER, sER, smooth ER, UPR, unfolded protein response, ERAD, ER-associated degradation, ULK, Unc51-like autophagy activating kinase, FIP200, FAK-family kinase interacting protein of 200 kDa, HSAN, hereditary sensory and autonomic neuropathy, FAM134B, family with sequence similarity 134, member B, LDS, LIR docking site, HP1/2, hydrophobic pocket ½, RETREG1, reticulophagy regulator 1, Atg11BR, Atg11-binding region, AIM, Atg8-interacting motif, NDP52, nuclear dot 52 kDa protein, LIR, LC3-interacting region, ATG, autophagy-related gene, ERAD, ER-associated degradation, UPR, unfolded protein response, TEX264, testis-expressed protein 264 precursor, CCPG1, cell cycle progression gene 1, RTN3L, reticulon 3 long isoform, SQSTM1, sequestosome 1, SEC62, translocation protein SEC62, ATZ, alpha-1-antitrypsin, NPC1, Niemann-Pick C1 protein, ATL, atlastin, MAP1LC3, microtubule associated protein 1 light chain 3, GABARAP, gamma aminobutyric acid receptor-associated protein, PI, phosphatidylinositol, GIMs, GABARAP-interacting motifs, FIR, FIP200-interacting region, RHD, reticulon homology domain, PC, procollagen, ERLAD, ER-to-lysosome associated degradation

## Abstract

The endoplasmic reticulum (ER) is a fundamental organelle in cellular metabolism and signal transduction. It is subject to complex, dynamic sculpting of morphology and composition. Degradation of ER content has an important role to play here. Indeed, a major emerging player in ER turnover is ER-phagy, the degradation of ER fragments by selective autophagy, particularly macroautophagy. This article proposes a number of unifying principles of ER-phagy mechanism and compares these with other selective autophagy pathways. A perspective on the likely roles of ER-phagy in determining cell fate is provided. Emerging related forms of intracellular catabolism of the ER or contents, including ER-phagy by microautophagy and selective ER protein removal via the lysosome, are outlined for comparison. Unresolved questions regarding the mechanism of ER-phagy and its significance in cellular and organismal health are put forward. This review concludes with a perspective on how this fundamental knowledge might inform future clinical developments.

## Highlights

• ER-phagy selectively degrades fragments of ER via the macroautophagy pathway.
• ER membrane localized cargo receptors bind the ER to core autophagy proteins.
• ER-phagy requires ER reshaping and may be selective for sub-ER content.
• Different receptors link stress signals to differential degradation of ER content.
• ER-phagy controls the pathogenesis of diseases including infection and cancer.

## Graphical abstract

**Figure:**
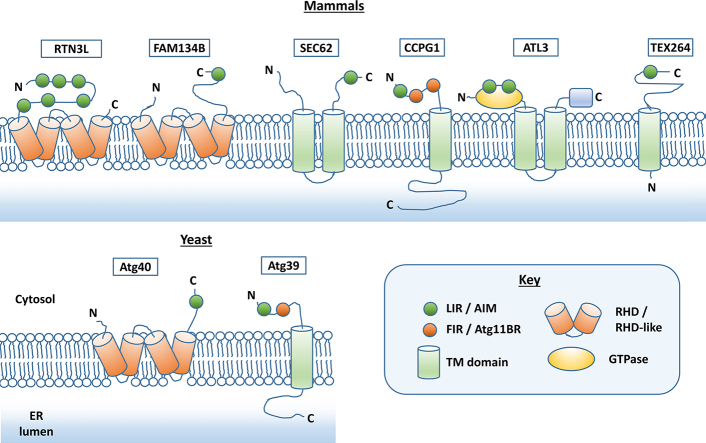

## Introduction

The endoplasmic reticulum (ER) is a single, continuous network of phospholipid bilayer delimited tubules and sheets [1]. It is found in all eukaryotes, from yeast through to mammals. The ER is divisible into distinct gross morphologic domains. The nuclear envelope domain is a spherical sheet, which gates the nucleoplasm from the cytoplasm. In contrast, the peripheral ER extends into the cytoplasm. In mammalian cells, this peripheral domain consists in part of the perinuclear sheet-like region, wherein fenestrated, flattened sacs, connected by helicodial tubes, stack against each other, in regularly spaced arrays [2], [3]. This region of the ER is the main site of protein synthesis, exhibiting extensive polyribosome attachment and has a distinct, studded appearance in ultrastructural analyses, leading to application of the term “rough ER” (rER). In the predominant mode of secretory protein synthesis, nascent polypeptides made by attached polyribosomes are inserted co-translationally into the ER lumen. These proteins fold inside the ER, protected from aggregation by lumenal chaperones, and form intra- and inter-molecular disulphide bonds catalysed by lumenal oxidoreductase enzymes. Nascent proteins are further modified by glycosylation catalysed by ER lumenal glycosyltransferases.

In addition to the rER, the peripheral ER also contains matrices of tubular ER, predominantly “smooth” ER (sER). Tubular ER is found in both the perinuclear and more distal regions of the cytoplasm [4]. These networks radiate out toward the plasma membrane in a characteristic pattern of three-way branches. sER is involved in metabolic functions of ER other than protein anabolism, such as phospholipid and steroid hormone synthesis, and detoxification. In yeast, the morphology of the ER is slightly different to mammals; the majority of the sheets and tubules of the peripheral ER are in close apposition to the plasma membrane and are termed the cortical ER.

The ER is also involved in cellular signaling. It acts as a sink for calcium, which is released into the cytosol in response to stimuli [5]. Furthermore, the cytosolic face of the ER membrane provides a residence for components of various signal transduction pathways [6], [7].

The ER membrane also platforms diverse protein complexes that facilitate contact with other cellular membranes, including mitochondria, endosomes, lipid droplets and the plasma membrane [8], [9]. These contacts facilitate regulation of organelle behavior, for example, mitochondrial metabolism and calcium homeostasis [10], and organelle trafficking and morphological rearrangement, the latter most notably within endocytic pathways [11], [12], [13].

The ER is highly specialized in certain cell types [14]. Thus, overall ER architecture and composition may be skewed strongly in favour of completing certain tasks. For example, the ER of skeletal muscle (sarcoplasmic reticulum) is a particularly extensive calcium store and controls the calcium waves that mediate myofibre contraction. The ER of steroid hormone producing cells, such as in the liver, or of digestive enzyme secreting exocrine cells, such as pancreatic acinar or gastric chief cells, is predominantly sER or rER, respectively.

Given the complexity of the ER in terms of its topology, composition and functional diversity, it is unsurprising that numerous cellular mechanisms exist to maintain the functional specialization and health of regions of the ER across different cellular contexts. The unfolded protein response (UPR) coordinates many of these mechanisms [14]. This homeostatic event is engaged in response to accumulated, partially folded protein within the ER lumen. It is a portfolio of signaling events triggered by the ER resident, membrane-embedded sensor proteins PERK (protein kinase R-like ER kinase), IRE1α (inositol-requiring enzyme 1 alpha) and ATF6 (activating transcription factor 6). The scope of this review does not extend to detailing the signaling cascades involved; excellent descriptions can be found in the literature [15], [16]. However, the result of these pathways is diminishment of global protein synthesis and thus import into the ER, with concomitant enhancement of ER capacity by changes in gene expression that drive ER expansion. These latter changes include increased lumenal chaperone and folding enzyme production. High-level, acute UPR signaling also engages cell death in sensitive cell types. Despite the prominence of the canonical UPR in the literature, it should be noted that other, less well-characterized stressors, such as lipid stress, which may engage the UPR or other signaling events, and less well-characterized signaling responses to ER stress, other than the UPR, have both been reported [17], [18], [19], [20].

Importantly, the UPR elevates cellular capacity for a degradative mechanism called ER-associated degradation (ERAD), which serves to retrotranslocate misfolded proteins from the lumen or membrane of the ER into the cytosol, whereupon they are ubiquitinated and proteasomally hydrolyzed [21]. The existence of ERAD illustrates a fundamental tenet of ER remodeling in cellular health, which is the core topic of this review: degradation of ER by molecularly targeted mechanisms. In particular, this review identifies and describes emerging principles of how the macroautophagy pathway mechanistically acts to isolate and select specific portions of ER for hydrolysis. This turnover of fragments of ER (membrane and lumenal contents) as cargo within sequestering vesicles known as autophagosomes, which then fuse with lysosomes (the vacuole in yeast), is known as macroER-phagy [14], or reticulophagy [22]. Although microautophagy pathways can also target ER to the lysosome or vacuole by direct engulfment (microER-phagy) [23], [24], [25], macroER-phagy shall be referred to as ER-phagy during the bulk of this review, for simplicity. Functionally, the cellular role of ER-phagy in some scenarios is to remove aberrant protein products from the lumen or ER membrane [26], [27], [28], [29], [30], [31], [32]. The mechanistic basis of this and the possibility of other cellular functions for ER-phagy are discussed herein. Furthermore, emerging data on the role of ER-phagy in maintenance of cellular and organismal health are considered, in order to illustrate its physiological importance. The text also briefly outlines other forms of selective lysosomal degradation of ER content that do not involve macroautophagy, such as microautophagy of the ER and other pathways with varying degrees of mechanistic overlap with ER-phagy. Finally, outstanding questions regarding the mechanisms and functions of ER-phagy, and the challenges in translating ER-phagy knowledge for human benefit, are presented.

## Core and Selective Macroautophagy

The canonical macroautophagy pathway (hereafter referred to simply as autophagy) is defined as the sequestration of material (cargo) from the cytoplasm into double-membrane vesicles called autophagosomes and subsequent degradation by fusion of autophagosomes with lysosomes. The movement of cytoplasmic material to the endpoint of this pathway, including eventual hydrolytic destruction in the lysosome, is referred to as autophagic flux [33]. In mammals, nascent autophagosomes form from a number of different membrane sources, the relative contribution of which is potentially dependent on the signaling pathways engaging the process. These membrane compartments may include plasma membrane, endosomes, ER and the Golgi apparatus [34]. Autophagosomes may initiate by deformation of an individual membrane compartment to provide the primitive double lipid bilayer tubular structure, known as the isolation membrane or phagophore, to which other membrane sources, in the form of vesicles, can fuse. Most notably, this has been characterized when the ER itself undergoes morphological alteration to form a so-called omegasomal structure, from which the phagophore protrudes as a thin membrane tubule. The phagophore has limited cross-sectional area and thus incorporates, at most, a modest amount of ER lumenal content, even assuming it remains continuous with the ER [35], [37], [38]. Any continuity with the ER is probably not maintained for long; recent studies show that lipid transfer from the ER to the growing phagophore occurs at tethers between discrete ER and phagophore membranes, via ATG2 lipid transfer proteins [39], [40]. Alternately, particularly during selective autophagy, it is possible that phagophore establishment is signaled *de novo* around cargo. In any event, expanding membranes eventually seal and are scissioned from the parental organelle prior to fusion with lysosomes. Both lysosomes and mature autophagosomes may subsequently be trafficked to bring the two compartments into proximity, facilitating fusion.

A number of largely evolutionarily conserved proteins (ATG, or *A*u*t*opha*g*y-related proteins) participate in the core steps of the macroautophagy pathway, regardless of the cargo being targeted. A brief outline of these key players and associated proteins in mammals is given here, in order to illuminate the remainder of the review; in-depth reviews devoted to the topic should be consulted for further information [34], [41]. Any relevant divergence in the core autophagy machinery between mammals and yeast will be highlighted throughout this review, where relevant to the overall topic of ER-phagy.

### Core mechanisms of autophagy

In the apical step of the canonical autophagy pathway, activation of a quadripartite serine–threonine kinase complex (the ULK complex), consisting of an active enzyme (ULK1/2, Unc51-like kinases) and three scaffold proteins, FIP200 (focal adhesion kinase-interacting protein 200 kDa), ATG13 [42] and ATG101 [43], results in phosphorylation of a number of downstream targets that promote autophagosome biogenesis [42], [44], [45], [46], [47]. Prominent among these targets is the class III phosphatidylinositol (PI)-3′-kinase complex, consisting of the lipid kinase subunit hVPS34 (vacuolar and protein sorting 34) and scaffolding or regulatory subunits, Beclin 1 (ATG6), ATG14 and VPS15. Phosphorylation of hVPS34 and Beclin 1 by ULK complexes results in hVPS34-mediated lipid phosphorylation of PI to form PI-3′-phosphate (PI3P) at nascent phagophores. In turn, this facilitates recruitment of PI3P binding proteins such as WIPI2 (WD repeat domain, phosphoinositide interacting 2) [48]. WIPI2 and FIP200 recruit ATG16L1, which also interacts directly with lipids [49], [50], [51]. ATG5–ATG12, a covalent conjugate of the C-terminal glycine of the ubiquitin-like protein ATG12 to a lysine on ATG5, is corecruited by ATG16L1. The tripartite ATG16L1–ATG5–ATG12 complex then acts as an E3 ubiquitin ligase-like enzyme to conjugate a family of GABARAP/LC3 (ATG8) ubiquitin-like protein paralogues to phosphatidylethanolamine lipid in the growing phagophore (lipidation). Note that the term GABARAP/LC3 is used throughout this review when referring collectively or non-specifically to mammalian member(s) of this family (MAP1LC3A, MAP1LC3B, MAP1LC3C, GABARAP, GABARAPL1, GABARAPL2/GATE-16). There is a sole Atg8 orthologue in yeast. GABARAP/LC3 lipidation is required for optimal autophagosome expansion or closure, although reduced autophagic flux may still occur when this is ablated [52], [53].

The final steps of autophagy involve encounter between autophagosomes and membranes of the endolysosomal pathway, including endosomes and multivesicular bodies [54], [55]. This ultimately leads to autophagosomal fusion, acidification and degradation of the sequestered cargo. Complementary SNARE (SNAP receptor) proteins on the outer autophagosomal membrane and on endolysosomal pathway membranes interact with each other to mediate fusion. The autophagosomal STX17 (syntaxin 17)–SNAP29 (soluble NSF attachment protein) complex mediates fusion to lysosomal membranes presenting surface VAMP8 (vesicle-associated membrane protein 8) [56]. This is assisted by additional tethering factors, including the interaction of another autophagosomal SNARE, YKT6, with lysosomal STX7, again via the SNAP29 intermediary [54], [55], [57]. In another example, fusion can be facilitated by GABARAP/LC3 on the outer autophagosomal membrane interacting directly with PLEKHM1 (pleckstrin homology domain containing, family M, member 1), which binds Rab8 on the cytosolic face of the lysosome [58].

### Selective mechanisms of autophagy

General autophagic flux is upregulated by nutrient responsive signals. Most notably, this occurs via regulation of ULK complex activity by inhibitory phosphorylation of ULK1/2 by mTORC1 serine–threonine kinase (mTOR complex 1), in response to amino acids, or by activating phosphorylation by the serine–threonine kinase AMPK (adenosine monophosphate-activated protein kinase) in response to elevated intracellular AMP/ADP to ATP ratios [59]. One outcome of upregulation of general autophagic flux is bulk, non-selective degradation of cytoplasmic material and generation of metabolites (amino acids, nucleotides, saccharides, lipids) to sustain metabolism and bridge nutrient hiatuses [60].

However, in selective autophagy, specific moieties within the cytoplasm are targeted for sequestration into autophagosomes and subsequent degradation, to the prominent exclusion of general cytoplasm [61], [62]. Selective clearance of mitochondria (mitophagy), cytoplasmic bacterial pathogens (xenophagy) and protein aggregates (aggrephagy) are important examples that are now mechanistically well-established in the literature. Usually, the purpose of selective autophagy is to remove a damaged or otherwise unwanted structure from the cytoplasmic environment. In this case, the sequestration into closed autophagosomes per se is perhaps the most important step for cell physiology. Nonetheless, in some instances, such as glycophagy (degradation of glycogen granules to make free glucose available in the liver, particularly in neonates), the completion of flux and the release of hydrolysis products is also critical [63], [64]. Notwithstanding such counterexamples, the key principle of selective autophagy is thus gathering of organellar or macromolecular protein complex cargo into the nascent autophagosome, prior to vesicle closure. These organelles and complexes are recognized by cargo receptors, which are bifunctional molecules that bind directly to the core autophagy machinery on the phagophore and nascent autophagosome, and directly or indirectly to cargo, but which are dispensable for stimulation of general, bulk autophagic flux [65]. Cargo receptors are also generally lysosomally degraded along with the cargo.

Frequently, cargo receptors interact directly with GABARAP/LC3, via a LIR (LC3-interacting region) motif, which is minimally a tetrapeptide sequence composed of a key bulky aromatic residue at position 1 and a key aliphatic residue at position 4, conforming to the consensus sequence [W/F/Y]XX[L/I/V] [66]. When this motif is exposed on the surface of a cargo receptor, hydrophobic pockets in GABARAP/LC3 envelop the two key residues. Yeast cargo receptors bind yeast Atg8 via a similar interaction (employing LIR-like Atg8-interacting motifs, abbreviated to AIMs) [67]. Furthermore, LIR motifs may be extended at the N-terminal side, presenting acidic residues or phosphorylatable serines and threonines. At cytosolic pH, the negative charge of acidic or phosphorylated residues permits binding to a positively charged surface region of GABARAP/LC3, strengthening interaction [69], [70], [71]. LIR motifs may be sub-classified into classical LIR motifs and GABARAP-interacting motifs (GIMs), based on preference for LC3 subfamily members (MAP1LC3A-C) or GABARAP subfamily members (GABARAP and GABARAPL1/2), respectively [68]. Furthermore, a non-canonical LIR motif that interacts specifically with MAP1LC3C has been described [72]. An emerging, additional mode of interaction of mammalian cargo receptors with the core autophagy machinery is between a so-called Atg11 homology region at the C-terminus of FIP200 (which has some sequence similarity to yeast Atg11), and FIP200-interacting region(s) (FIRs) on cargo receptors. Yeast cargo receptors, on the other hand, frequently bind Atg11 via Atg11-binding regions (Atg11BRs) [67]. Currently, mammalian FIRs are imprecisely defined, having been found on only three mammalian cargo receptors and two cargo receptor-binding adaptor proteins [30], [73], [74], [75]. However, some may have a core similarity to yeast Atg11BRs, which are composed of a di-aliphatic sequence surrounded by serine, threonine-, aspartate- and glutamate-rich sequence. Finally, receptors can be multivalent for the autophagy machinery, containing multiple LIRs, or LIR(s) and FIR(s), within the same polypeptide sequence.

Regardless of the mode of interaction, receptors are recruited to aberrant or surplus cellular structures earmarked for degradation as autophagy cargo. The target of molecular recognition of these cargoes by receptors may be protein post-translational modifications. For example, aggregating proteins or surface proteins of some organellar cargoes to be degraded are modified with polyubiquitin [76]. Broken phagocytic or endolysosomal vesicles expose ordinarily lumenal β-galactoside carbohydrate moieties to the cytosol, which in turn recruit cytosolic carbohydrate-binding proteins such as galectin-3 or − 8 [77], [78]. Both polyubiquitin and galectins are recognized via binding domains on cargo receptors. Molecular recognition of cargo can also be stimulated independently of recognition of ubiquitin or carbohydrate, for example, by exposure of receptor binding proteins (or even lipid species) that are ordinarily shielded from the cytoplasm in response to stress or damage [79], [80]. Signaling might also impinge on selective autophagy flux capacity in response to cognate stresses, for example, by upregulation of cargo receptor expression at a transcriptional level such as occurs in response to hypoxia for the mitophagy receptors BNIP3 (Bcl2-interacting protein 3) and BNIP3L (BNIP3-like) [81], [82]. Finally, while the unifying principle of cargo receptor function is that they link cargo to nascent autophagosomes, some receptors may also play an active role in stimulating the generation of autophagosomes around cargo. For example, the recruitment of FIP200-containing ULK complexes to mitochondria or bacteria (via direct binding of FIP200 by the cargo receptor NDP52, nuclear dot protein 52), may locally initiate the autophagy process, with MAP1LC3C binding by NDP52 occurring subsequently [73], [75]. In another example, FIP200 recruitment by the receptor p62/SQSTM1 (sequestosome 1) in turn draws ATG16L1 to protein aggregates, in order to promote autophagy [74]. Altogether, selective autophagy is thus a regulated molecular program that results in targeted incorporation of specific cargoes into autophagosomes.

## Molecular Principles of ER-Phagy

ER-phagy is an emerging form of selective macroautophagy that uses cargo receptors to facilitate degradation of portions of ER. Befitting the complexity and heterogeneous functions of the ER, it is important to note that ER-phagy may be an umbrella term for multiple, conceptually similar pathways of selective autophagy, differing at the level of mechanistic detail and cellular purpose. This section of the review thus attempts to delineate some emerging, unifying principles of ER-phagy mechanism, drawing parallels with broader tenets established by study of other forms of selective autophagy. Notably, this comparative approach also highlights some relatively unique challenges that ER cargo presents, such as generating degradable fragments from within the network, and targeting degradation to specific network subregions. This framework for conceptualizing and exploring ER-phagy provides an alternative to that presented in some other recent reviews, which explore ER-phagy receptor function on a molecule-by-molecule basis [83], [84]. The interested reader is encouraged to consult these.

### Principle 1: Recognition of ATG proteins by ER-resident cargo receptors

**Fig. 1:**
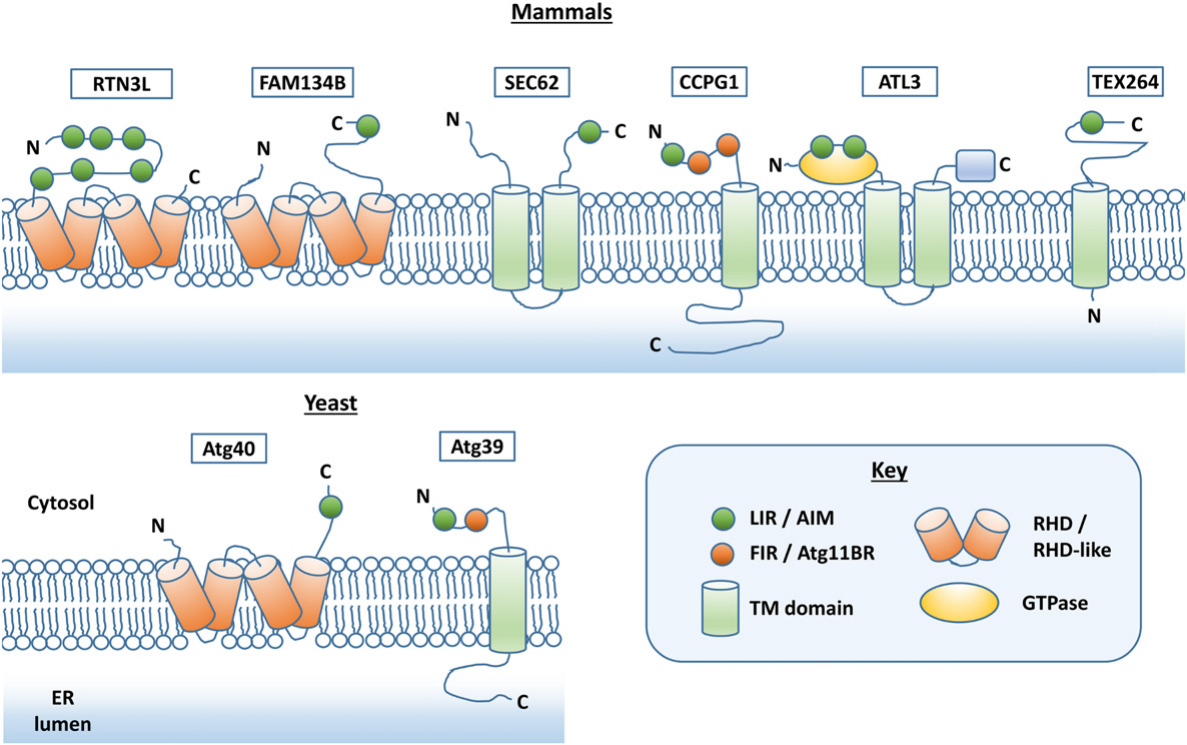
Structure of ER-phagy cargo receptors in mammals and yeast. Abbreviations: LIR, LC3-interacting region (mammals); AIM, Atg8-interacting motif (yeast equivalent of LIR); FIR, FIP200-interacting region (mammals); Atg11BR, Atg11-binding region (putative yeast equivalent of FIR); TM, transmembrane; GTPase, dynamin-like GTPase domain; RHD, reticulon-homology domain (mammals); RHD-like, putative reticulon-homology domain like structure (yeast).

As with other forms of selective macroautophagy, the key characteristic of ER-phagy is the involvement of cargo receptor molecules. In principle, these could be ordinarily soluble cytosolic molecules that would bind to modified or novel protein complexes resident in the ER membrane, and directly to ATG proteins. However, to date, the most well-characterized ER-phagy receptors (six in mammals and two in yeast) are either directly ER membrane anchored, via insertion of part of the polypeptide into the ER membrane from the cytosolic side, or are bona fide transmembrane proteins (Fig. 1). Table 1 provides a summary of the characteristics of these receptors that should be consulted throughout reading of this review.

**Table 1:** ER-phagy cargo receptors and functions

	FAM134B	RTN3L (RTN3 isoform e)	SEC62	CCPG1	ATL3	TEX264	Atg39 (*S.c.*)	Atg40 (*S.c.*)
Canonical isoform (NP_)	001030022	001252518	003253	004739	056274	001123356	013415	014795
Size (a.a.)	497	1032	399	757	541	313	398	256
LIR/AIM	455-FELL-468	217-YSKV-220 248-FEVI-251 342-WDLV-345 555-FEEL-558 790-YDIL-793	363-FEMI-366	14-WTVI-17	192-YGRL-195 390-FKQL-393	273-FEEL-276	8-WNLV-11	242-YDFM-245
FIR/Atg11BR				18-SHEGSDIEMLNS-29 101-SDDSDIVTLE-110			52-DVLS NTSS-59	
ER sub-region	Sheet	Tubular			192-YGRL-195 390-FKQL-393	Tubular (3-way junctions)	Nuclear envelope	Peripheral
ER anchor	RHD	RHD	Two TM	Single pass TM	Two TM	Single pass TM	Single pass TM	RHD-like
Membrane reshaping activity?	RHD	RHD			Dynamin-like GTPase			RHD-like
Stimulus	Nutrient starvation	Nutrient starvation	Recovery from UPR	UPR	Nutrient starvation	Nutrient starvation	Rapamycin	Rapamycin
Protein targets in ER-phagy?	PC clearance Mutant NPC1	Small role in PC clearance?	UPR induced chaperones	Exocrine enzymes & chaperones in pancreas Role in PC clearance?		Broad rang Selectivity evident		
Altered in disease?	Expression (HSAN II)		Amplified (cancer)		Point mutation (HSAN I)		n/a	n/a

Summary of the characteristics of the eight well-characterized ER-phagy receptors discussed in this review. All proteins are human isoforms except for those marked (*S.c.*) for yeast *Saccharomyces cerevisiae*. Abbreviations: a.a., amino acids; RHD, reticulon-homology domain; TM, transmembrane; HSAN, hereditary sensory neuropathy; UPR, unfolded protein response; PC, procollagen; ERLAD, ER-to-lysosome associated degradation; NP, NCBI Reference Sequence prefix. n/a, not applicable.

All known ER-phagy receptors contain at least one LIR motif or AIM, and thus bind to GABARAP/LC3 family proteins (mammals) or Atg8 (yeast). The first mammalian receptor to be discovered was FAM134B (family with sequence similarity 134, member B), also known as RETREG1 (reticulophagy regulator 1) [85]. There is a reticulon homology domain (RHD) toward the N-terminal end of FAM134B that tethers FAM134B to the ER membrane. RHDs are tandem helical hairpin structures that mediate insertion into the ER membrane from the cytosolic face [86]. The N- and C-termini of FAM134B are cytosolic and GABARAP/LC3 recognition is encoded in a single C-terminal LIR motif (core sequence FELL in humans). Interestingly, the FAM134B sequence paralogues FAM134A and FAM134C also bind GABARAP/LC3, but their potential involvement in ER-phagy is not yet known [85]. A possible structural orthologue of FAM134B is found in yeast ER-phagy pathways in the form of Atg40, which has an RHD-like domain at its N-terminus and an AIM at its C-terminus (core sequence YDFM) [87]. A second RHD-containing receptor in mammals, RTN3L (reticulon 3 long), is a long splice isoform of a ubiquitous reticulon protein, RTN3 [88]. RTN3 isoforms have a C-terminal RHD that mediates anchoring to the ER from the cytosolic face of the membrane. The RTN3L isoform has an extended N-terminus that contains six distinct LIR motifs, spaced at uneven intervals (core sequences from N-terminus to C-terminus: FTLL, YSKV, FEVI, WDLV, FEEL, YDIL). Mammalian SEC62 (secretory 62 homologue) is also a cargo receptor [27]. This protein ordinarily participates in post-translational import of protein into the ER. It is incorporated into the ER membrane via two transmembrane domains, which are linked by an ER lumenal peptide, and it has cytosolic N-terminal and C-terminal regions. The cytosolic C-terminal region of SEC62 contains a single LIR (core sequence FEMI). Interestingly, the yeast orthologue Sec62, while participating in protein import into the ER, does not contain an AIM and does not have a role in ER-phagy [27].

**Fig. 2:**
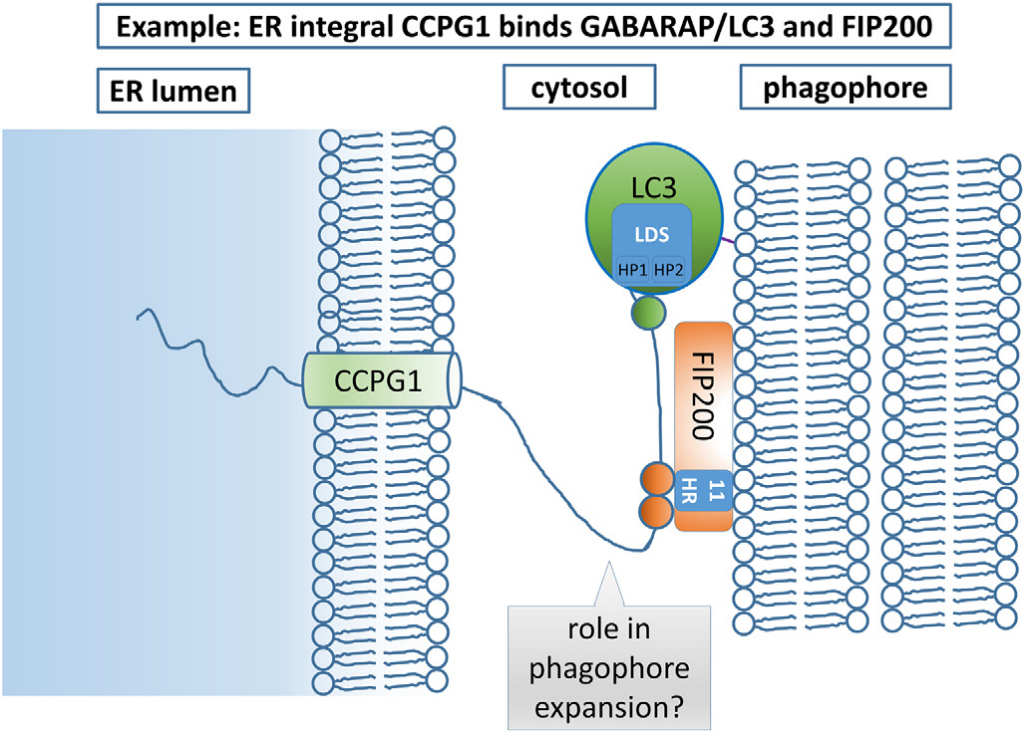
Principle 1: Membrane integral receptor proteins bind to the phagophore. This is exemplified here by consideration of the mammalian cargo receptor CCPG1, which illustrates a number of key points. It is embedded in the single phospholipid bilayer of the ER (membrane on left hand side of cartoon) via a single transmembrane domain. It also binds to ATG proteins assumed resident on the phagophore (growing double phospholipid bilayer, right-hand side of cartoon). First, lipid-conjugated GABARAP/LC3 family members contain a LIR docking site (LDS), which is home to the two hydrophobic pockets (HP1 and HP2) that accommodate the hydrophobic LIR motif on CCPG1 (green circle). All known ER-phagy cargo receptors contain one or more LIR motifs for binding GABARAP/LC3 (or AIM motifs for binding Atg8 in yeast). A second interaction, seen with only some cargo receptors such as CCPG1 or Atg39, is direct binding of a FIR motif(s) (orange circles) to the Atg11 homology region (Atg11HR) at the C-terminus of FIP200 (the Atg11BR, or Atg11-binding region, of yeast Atg39 binds to the autophagy protein Atg11 in an analogous interaction). CCPG1 is depicted here as having two discrete FIR motifs interacting with one molecule of FIP200, but it must be noted that the precise structural details of this interaction, and definition of what constitutes a single mammalian FIR motif, are not yet known. The cytosolic region of CCPG1 is intrinsically disordered, potentially allowing sufficient distance between the GABARAP/LC3 and/or FIP200 interaction sites from the outer leaflet of the ER to avoid steric hindrance (as experimentally demonstrated for TEX264). In principle, receptors need not be ER membrane integral proteins but could form a complex with integral proteins. However, the best-characterized receptors to date (Fig. 1) are all anchored in the ER membrane. Minimally, receptors link cargo to the phagophore or growing autophagosome. However, it is emerging that some, particular those that bind FIP200 and thus potentially the ULK complex, such as CCPG1, might also influence the formation or growth of the phagophore. See “Function and interplay of ATG protein interactions” for further discussion of this.

More recently discovered ER-phagy receptors in mammals include CCPG1 (Fig. 2), a single transmembrane domain protein that harbors an extensive C-terminal ER lumenal region of undefined structure and an intrinsically disordered N-terminal cytosolic region [89]. CCPG1 contains a single LIR motif at the extreme N-terminus (core sequence WTVI) [30], [31], [32]. In addition to the LIR motif, CCPG1 also links the ER to the autophagy apparatus via two FIR motifs (SHEGSDIEMLNS and SDDSDIVTLE), the former being localized adjacent to the LIR motif and the latter being further C-terminal. The latter also makes the most significant contribution to FIP200 binding, consistent with a more concentrated field of negative charge and potentially phosphorylatable residues. CCPG1 is the only mammalian ER-phagy receptor with this dual GABARAP/LC3 and FIP200-binding property, the other two mammalian cargo receptors with this property being the cytosolic ubiquitin-binding proteins NDP52 and p62/SQSTM1 [73], [74], [75]. Discrete mechanistic functions of these two different interactions of CCPG1 are not yet known, but both appear equally important for ER-phagy [30]. Despite CCPG1 having no direct sequence orthologues outside vertebrates, the ER-phagy receptor Atg39 is nevertheless a potential CCPG1 structural orthologue in yeast [87]. Atg39 is a single pass transmembrane protein with a cytosolic N-terminus and a lumenal C-terminus with an N-terminal AIM (core sequence WNLV) and Atg11BR (DVLSNTSS).

Another recently discovered, single transmembrane domain ER-phagy receptor is TEX264 (testis-expressed protein 264) [90], [91]. TEX264 has a negligible N-terminal lumenal region of approximately 5 amino acids and an extensive, approximately 286-amino-acid C-terminal cytosolic region. TEX264 has a single LIR (core sequence FEEL) near the C-terminus [90], [91]. The LIR interacts preferentially with MAP1LC3A, GABARAP and GABARAPL1 [91]. While the TEX264 cytosolic region is partially structured, the C-terminal 113 amino acids, encompassing the LIR, are intrinsically disordered. Interestingly, the precise amino acid sequence of this region, other than the LIR, may be irrelevant to the participation of TEX264 in ER-phagy, as long as the polypeptide is of sufficient flexibility and length [91]. These observations led to the proposition that the intrinsically disordered region of TEX264 is a spacer, which ensures that interactions with the autophagy machinery at the growing phagophore are sufficiently distant from the cytosolic face of the ER to avoid steric hindrance by other macromolecular assemblies, for example, polyribosomes. Interestingly, regions of intrinsic disorder encompassing the LIRs and FIRs of other mammalian receptors have been identified, that is, within CCPG1, FAM134B, RTN3L and SEC62 [91].

The final example of an ER-phagy receptor in mammals is ATL3 (Atlastin 3) [92]. It has two transmembrane regions connected by a lumenal polypeptide region. Unlike the other mammalian receptors, the LIRs (core sequences YGRL and KQKL, respectively) are not located within an intrinsically disordered region, being present within a cytosolic, N-terminal dynamin-like GTPase domain. The ATL3 LIRs are specifically GIMs, as ATL3 preferentially binds to GABARAP subfamily members of the GABARAP/LC3 family. Note that there is as of yet no example of a non-canonical LIR motif, such as the MAP1LC3C-binding motif of NDP52 [72], being employed among mammalian ER-phagy receptors.

It should be noted that candidates for membrane peripheral ER-phagy receptors (i.e., non-integral) have been proposed, but these need further investigation. For example, p62/SQSTM1 is present on ER fragments contained within autophagosomes implicated in basal ER turnover in mouse liver, and in elevated turnover induced by 1,4-bis[2-(3,5-dichloropyridyloxy)] benzene toxicity [93]. However, the involvement of p62/SQTSM1 in mediating ER-phagy per se is not yet demonstrated. Nonetheless, p62/SQSTM1 can bind to ER membrane integral IRE1α, suggesting how function as an ER-phagy cargo receptor function could hypothetically be fulfilled [94]. The ER lumenal chaperone protein calreticulin contains a LIR motif that binds GABARAP/LC3 *in vitro* [95]. However, it is unclear how this would interact with cytosolic GABARAP/LC3 *in cellulo*. Finally, BNIP3 is a LIR-motif containing mitophagy receptor. Overexpression of a chimeric form of BNIP3 attached to an ER localization sequence can drive ER-phagy but is unclear whether this experiment models a physiologic process [96].

### Principle 2: ER linkage to the phagophore may be co-ordinated with ER reshaping

**Fig. 3:**
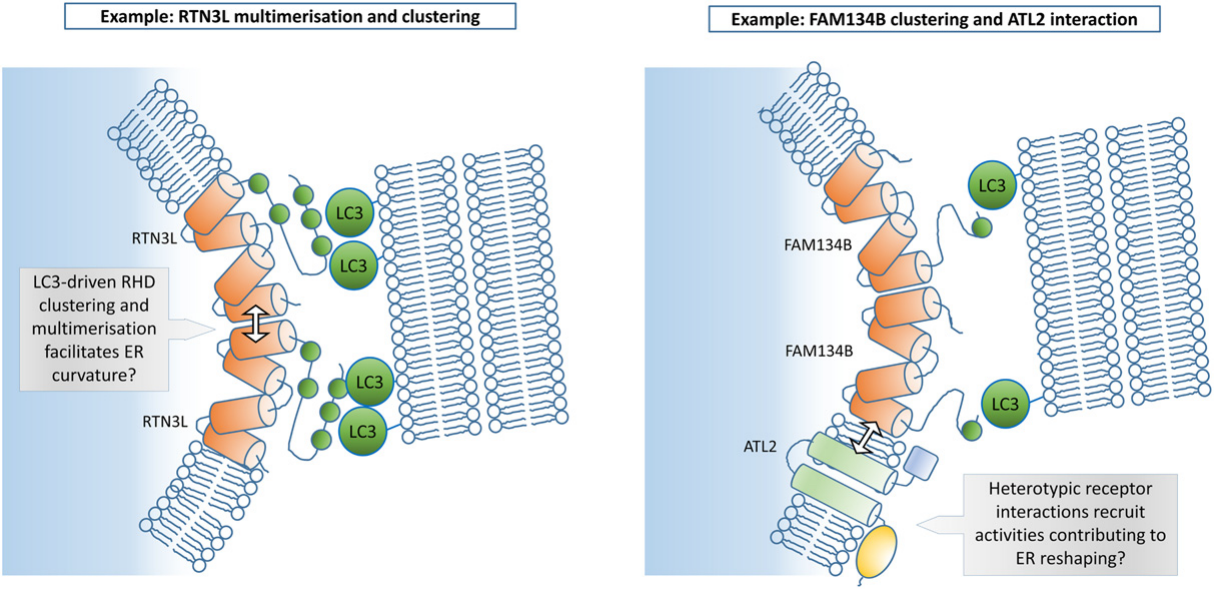
Principle 2: Receptors cluster and bind other proteins at initiation sites to drive ER reshaping. This is exemplified by studies of RTN3L and FAM134B. RTN3L has a reticulon-homology domain (RHD; represented by four orange cylinders) that drives membrane curvature. RTN3L clustering and deformation of ER membrane can be facilitated by GABARAP/LC3 interaction at the phagophore via LIR motifs (green circles) and also by homo-oligomerisation. It is unknown whether RTN3L interacts heterotypically with other receptors or ancillary membrane-reshaping proteins to drive tubulation and bending of the ER at phagophores and into nascent autophagosomes. Similarly, FAM134B has an RHD. It can cluster dependent on GABARAP/LC3 interaction via its sole LIR motif. Heterotypic interaction of FAM134B with the transmembrane GTPase and ER morphology factor ATL2 may be important for reshaping required for ER-phagy (ATL2 GTPase domain represented by yellow oval). However, other receptors, for example, SEC62 or CCPG1, have no obvious domains that would drive membrane reshaping, and it is likely ER reshaping is driven by other molecules at autophagy initiation sites, possibly recruited by direct or indirect interaction with the receptor itself. In principle, these molecules could encompass non-ATG binding ancillary proteins and/or other RHD-containing or dynamin-like GTPase domain-containing receptors.

Some targets of selective autophagy, such as bacteria or protein aggregates, are encapsulated whole by the autophagosome, which has a typical diameter of between 0.5–1.5 μm in mammals [97]. However, the ER is a continuous structure that occupies a large proportion of the volume of the cell. Thus, in ER-phagy pathways, the ER must be fragmented at some point prior to closure of the growing autophagosome. In some pathways, it is hypothetically possible that discrete fragments of ER will be generated prior to recruitment of ATG proteins and stimulation of local phagophore formation and growth. However, the data that are available thus far suggest that the ER membrane to be degraded interacts with autophagosomal membranes prior to fragmentation. MAP1LC3A localizes to foci at three-way junctions of the tubular ER, prior to LIR motif-dependent TEX264 recruitment [90]. Some foci of TEX264 recruitment also colocalize with phagophore markers FIP200 and WIPI2 [91]. Furthermore, in ultrastructural studies, TEX264-positive ER membranes were detected in cross-section as tubules found in close apposition to, and curved around the perimeter of, the inner autophagosomal membrane [90]. Taken together, these data suggest that ER membrane remodeling might lead to formation of a tubular extrusion and/or curving of an existing tubule concomitant with binding of this structure to the nascent autophagosomal membrane via GABARAP/LC3-TEX264 interaction. This ER structure would be scissioned prior to autophagosome closure, by mechanisms potentially involving generation of discrete “rings” of ER, as appear to be detected by TEX264 microscopy [90]. Membrane reshaping activities driving such ER remodeling might, in some instances, require receptors themselves; for example, when those receptors with intrinsic membrane deforming activity cluster locally, or when receptors bind and recruit other membrane reshaping proteins (Fig. 3). The RHD found in FAM134B and RTN3L inserts asymmetrically into the ER membrane from the cytosolic side, pushing the outer leaflet of the lipid bilayer apart [86]. Thus, when FAM134B and RTN3L cluster, this would be predicted to drive membrane curvature. Indeed, ectopic expression of FAM134B in cells basally, or RTN3L in nutrient-starved cells, shows that these proteins will cluster at ER-phagy initiation sites and drive punctation of the ER into autophagosomes [85], [88]. Importantly, ectopic expression of FAM134B or RTN3L bearing mutated LIR motifs in these studies shows that GABARAP/LC3-binding is critical for this activity [85], [88]. Supporting this, FAM134B- and RTN3L-driven ER punctation was shown to be dependent on ATG5 and ATG7, respectively (which are upstream of GABARAP/LC3 lipidation). These data strongly imply that interaction with membrane-tethered GABARAP/LC3 is necessary to cluster FAM134B and RTN3L, not only to mediate linkage of ER to the phagophore but to concomitantly drive membrane reshaping for packaging into autophagosomes. Notably, the ATL3 receptor can homodimerize, but the significance of this in ER-phagy has not been addressed [98].

Protein–protein interactions other than that of core ATG proteins with receptors are involved in this process. For instance, RTN3 isoforms multimerize and, indeed, artificial homo-multimerization of ectopically expressed RTN3L protein, using a rapalogue inducible system, is sufficient to drive ER punctation into autophagosomes in unstarved cells [88]. This suggests that RTN3L self-interaction drives clustering and ER reshaping. In another example, ER-phagy may also involve the membrane reshaping activity of the Atlastin proteins, ATL1–3 [99]. While ATL3 is a *bona-fide*, LIR (GIM)-containing cargo receptor, all three Atlastins have a similar overall structure with a cytosolic, N-terminal dynamin-like GTPase domain that promotes ER budding, and could potentially also drive final scission events, at least in concert with other reshaping activities. It is notable in this regard that ATL2 may bind FAM134B and is required for FAM134B-driven autophagy, suggesting that these proteins interact and co-operate in ER-phagy [99].

In summary, some receptors have intrinsic membrane reshaping activity that may be required for ER-phagy, which is co-ordinated with phagophore and nascent autophagosome recognition by ATG protein binding and clustering. Receptors may also self-interact, potentially interact with one another, or interact with other molecules with membrane reshaping activity in order to co-ordinate the morphological rearrangements of ER required for packaging into autophagosomes. Notably, this mechanism may be unique to ER-phagy; parallels in other selective autophagy pathways are not readily apparent. For example, while elongated, fused mitochondria may be protected from mitophagy [100], there is no evidence that mitochondrial fission processes are co-ordinated by mitophagy cargo receptors.

### Principle 3: Receptors mediate selection of subregions of the ER, or content, for degradation

The ER is not homogenous throughout the network and is subject to local fluctuations in homeostasis. Thus, ER-phagy is very likely co-ordinated with targeting of individual regions of ER for degradation. This was evident in yeast studies that showed that Atg39 predominantly removed the nuclear ER (nuclear envelope) and Atg40 the cytosolic and cortical ER (equivalent to the mammalian peripheral ER) [87]. In mammals, no nuclear membrane specific receptor has yet been described, but the sheet-like ER has been shown to be predominantly a target of FAM134B, while the tubular ER was shown to be degraded by RTN3L and ATL3 [85], [88], [92]. This may simply be a case of the steady-state localization of the receptors, given that RTN proteins generate curved regions of ER, so are by definition present at higher density on the tubular ER [102]. In contrast, they may only be found at the edges, and regions of fenestration, within sheet-like ER [4], [103]. It is possible this non-redundancy between receptors that reside in different regions of the ER has evolved simply to ensure coverage of the entire ER network with responsive ER-phagy pathways. However, this simple model of de facto targeting of different types of ER by differentially localized receptors is complicated by recent reports that TEX264 shows extensive colocalization with the apparent sheet-like ER receptor FAM134B at three-way junctions of the tubular ER [90]. As an alternative model, some receptors might be dynamically targeted to specific ER subregions for ER-phagy, leading to selective degradation of these regions and, by extension, apparent selectivity for particular ER proteins. In this vein, stimulation of ER-phagy may also require active transport of receptors through the ER network to specific regions of action, as demonstrated by the Lunapark- and actin-dependent transport of cortical Atg40 to the perinuclear subregion in yeast [104].

**Fig. 4:**
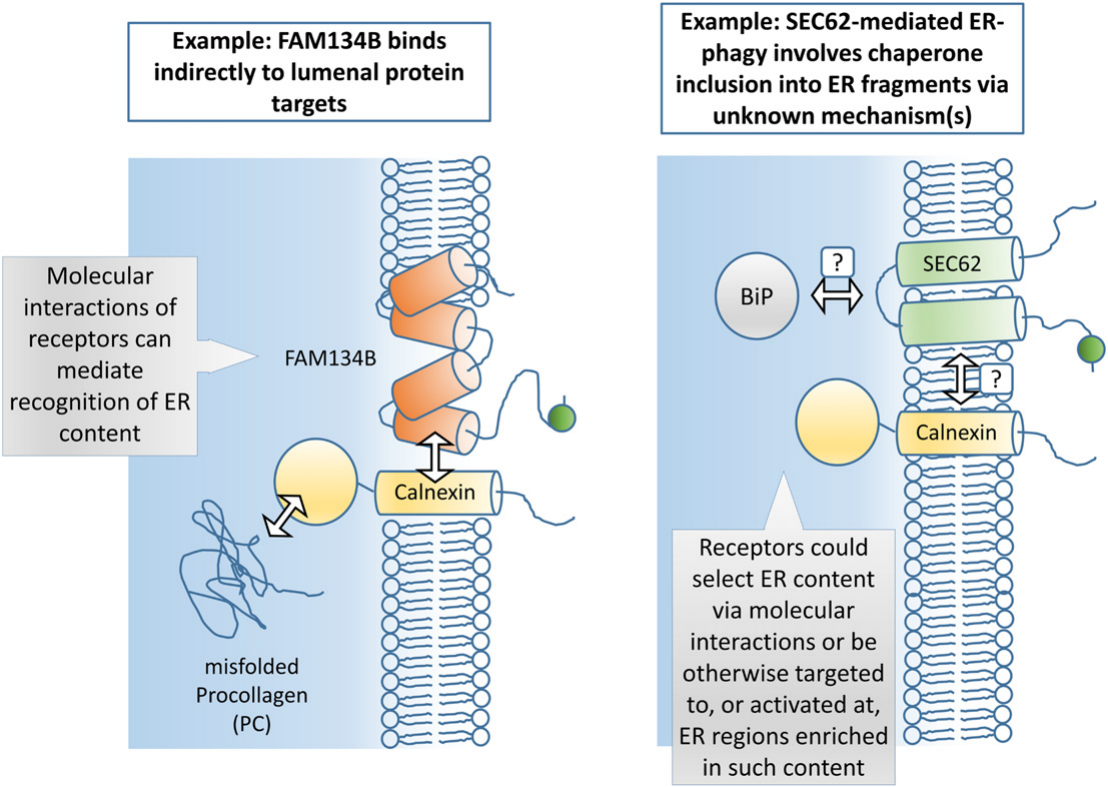
Principle 3: Selection of specific content from within the ER network for degradation. Selective degradation of ER content by ER-phagy may occur because receptors are recruited to the pathway at particular subER locales. Alternatively, and not mutually exclusively with such a mechanism, FAM134B provides an example of how molecular interactions can bridge receptors to lumenal cargo that is to be cleared preferentially from within the ER. Interaction of the RHD (orange cylinders) of FAM134B with the transmembrane domain of Calnexin enables indirect interaction of FAM134B with misfolded procollagen (PC), via the chaperone domain of Calnexin (yellow circle). Thus, FAM134B-driven ER-phagy can be biased toward fragments of ER that are heavily enriched in PC. In principle, other receptors, dependent on their domain structure, could participate in direct or indirect interactions with specific ER protein species that are localized either in the membrane or in the lumen. However, this area of ER-phagy study is in its infancy. In an alternative example, where the molecular mechanism is largely unclear, SEC62 promotes clearance of ER fragments enriched in UPR-upregulated chaperones, such as the integral membrane protein Calnexin and the lumenal protein BiP (Binding immunoglobulin protein). It is unclear whether SEC62 is activated locally, at regions where these cargo molecules accumulate (for example by loss of interaction with SEC63, see “ER-phagy is regulated by cellular state and signal transduction”), or whether SEC62 directly or indirectly interacts with the protein targets. These mechanisms are not mutually exclusive.

At a sub-ER level, emerging evidence suggests that one of the functions of ER-phagy is to target aggregation-prone protein species selectively into the sequestered fragments of ER. In particular, it has been shown that misfolded procollagen (PC) in the ER is cleared by FAM134B-dependent ER-phagy (Fig. 4). In this mechanism, there is indirect interaction of the cargo receptor with PC [26]. The membrane-resident segment of the transmembrane protein calnexin interacts with FAM134B; the lumenally resident portion of calnexin has a chaperone activity and binds PC. Thus, the receptor FAM134B not only links the ER to the ATG protein machinery but specifically incorporates lumenal protein species that require preferential clearance (or, vice versa, FAM134B is recruited to regions where lumenal protein species destined for degradation are already assembling into aggregates). Intriguingly, RTN3L and CCPG1 might also have minor roles in PC clearance according to data from this study, although the mechanistic basis of this was not examined [26]. PC may also be cleared by non-macroautophagy forms of ER-phagy [24] (discussed further in section “Relation of ER-phagy to other selective ER-to-lysosome degradation pathways”). Other misfolded proteins, such as the disease-associated I1061T variant of the transmembrane protein NPC1 (Niemman–Pick type C disease protein 1), may also be cleared by ER-phagy in a FAM134B-dependent manner (particularly when the default pathway of ERAD is compromised), although the molecular mechanism of recognition is unclear here [29]. During recovery from the UPR, cells remove excess ER containing chaperones that were upregulated during the stress response (Fig. 4). This has been termed recovER-phagy and is predominantly dependent on SEC62 action [27]. It is mechanistically unclear how SEC62 targets ER selectively enriched in these chaperones. Finally, nutrient starvation induces a turnover of ER dependent majorly on TEX264 in cultured cells, with significant contributions from FAM134B and CCPG1 [90], [91]. Here, unbiased proteomic profiling identified degradation of a cohort of over 300 ER proteins. While about half of this turnover was dependent on TEX264, the degree of TEX264 dependency was not equally distributed across the cohort, suggesting selectivity of TEX264 action. This observation is consistent with either of the two concepts outlined above; sub-ER localization properties of TEX264 might lead to preferential turnover of specific regions, or TEX264 might indirectly interact with proteins that are selectively degraded [90]. These models are not mutually exclusive.

Some distant parallels for selection of subregions of the ER for degradation may come from other selective autophagy pathways such as mitophagy, where, similarly, a large network structure may have to signal defects within a localized region to the autophagy apparatus. In particular, expression of mutant ornithine transcarbamylase in the mitochondrial matrix leads to protein aggregates and localized recruitment of the autophagy apparatus from the cytosol [105].

### Principle 4: ER-phagy is regulated by cellular state and signal transduction

Some forms of ER-phagy operate basal ER turnover in some cell and tissue types. For example, *Fam134b* null mouse embryonic fibroblasts (MEFs), or human U2OS cells knocked down for *FAM134B*, exhibit expanded ER [85]. However, perturbations of cell state can increase the ER-phagy flux dependent on given receptors, in cultured cells at least. For instance, nutrient starvation strongly upregulates autophagic flux in MEFs or simian COS7 cells. Several autophagy pathways are enhanced indiscriminately by this approach. For example, turnover of MAP1LC3B and the cytosolic receptor p62/SQSTM1 occurs, alongside sequestration of ER into autophagosomes and turnover in the lysosome, all dependent on the core autophagy machinery. However, loss of FAM134B or RTN3 protein in MEFs, ATL3 in COS7 cells, or TEX264, CCPG1 and/or FAM13B in HeLa or HCT116 cells, selectively prevents turnover of ER while leaving MAP1LC3B and p62/SQSTM1 degradation unperturbed [85], [88], [90], [91], [92]. It is possible that nutrient starvation upregulates all forms of autophagy, via mTORC1-dependent ULK complex regulation, or that it has discrete effects on the ER or ER-phagy pathways via unique signaling events. In yeast, Atg39 and Atg40-driven pathways are engaged by rapamycin, which mimics nitrogen starvation by inhibiting yeast TORC1 [87].

ER-phagy engagement events in some other settings are co-ordinated with cellular state via more overtly ER-centric signal transduction. For example, although CCPG1 modestly contributes to starvation-induced ER-phagy, wherein FAM134B is another significant player and TEX264 may exert the major effect [91], CCPG1 itself is induced by UPR-mediated transcriptional activation when cells are treated with ER stressors [30]. In particular, incorporation of the ER into autophagosomes and subsequent turnover is dependent on CCPG1 when HeLa cells are treated with the reducing agent dithiothreitol (DTT), which prevents protein folding in the ER lumen by interfering with disulphide bond formation and rearrangement, triggering the UPR. Furthermore, it is likely that signaling cascades set in train by the UPR play a role in priming SEC62-dependent recovER-phagy during the resolution phase. Interestingly, SEC62 participation in post-translational protein import, which occurs as a partnership with SEC63 and the SEC61 translocon complex, is mutually exclusive with a role in ER-phagy due to a competing interaction of SEC63 for SEC62 that blocks GABARAP/LC3 binding [27]. Cell signaling events likely determine this choice of function for SEC62, although these remain to be identified.

Modes of signaling involved in other selective organelle autophagy pathways, but not yet described for ER-phagy, are the regulated cytoplasmic exposure of receptor-binding proteins, or lipids, from within organelles, and the post-translational modification of cytosolic regions of organelle localized proteins.

## ER Functions in Health and Disease

Overall, the data described above have pointed to discrete functions of ER-phagy in cellular proteostasis and remodeling of the ER proteome. However, given the diversity of functions of the ER, which is not limited to protein production and secretion, it is likely other roles for ER-phagy will emerge, both within proteostasis and beyond. One way to gain further insight into this is to consider the effect of pathway disruption on the overall phenotype of cells and organisms. The recent discovery of ER-phagy receptors provides an excellent resource to interrogate such effects.

In cultured human and mouse cells, FAM134B protein plays a role in protecting against ER stressor-induced cell death [85]. *In vivo*, *Fam134b* knockout results in swelling of the ER in peripheral sensory neurons. These secretory cells undergo cell death, mimicking the phenotype of a human inherited disease, hereditary sensory and autonomic neuropathy (HSAN type II) [85]. This disease is associated with *FAM134B* mutations that result in premature translational termination and loss of GABARAP/LC3 binding by FAM134B, and likely nonsense-mediated decay of the *FAM134B* transcript [106]. Thus, in one particular cell type, in otherwise unstressed mammals, FAM134B plays a key role in regulating cell health. It is tempting to speculate that this relates to the role of FAM134B in PC proteostasis [26]. The *Fam134b* knockout mouse and HSAN type II patient samples should allow testing of this proposition. Surprisingly, *Rtn3*-deficient mice have no obvious defects in ER function, so the *in vivo* role of RTN3L-mediated autophagy remains undiscovered [107]. Interestingly, inherited mutations in *ATL3* in the first LIR (Y to C at position 1 of the motif, Y192C) and elsewhere in the protein (P338R) inhibit binding to GABARAP and result in a peripheral neurodegenerative disorder named HSAN type I, which has a pathology related to that of the FAM134B-associated HSAN type II [92]. This observation implies that ER-phagy is similarly involved here. However, a note of caution comes from the fact that these mutation(s) inhibit both the dimerization of ATL3, independently of GABARAP/LC3-binding, and other functions of ATL3 in ER organisation that are not necessarily intertwined with ER-phagy, such as regulation of ER export site abundance [98], [101]. ATL1 function is also ablated via inherited mutation in a degenerative disorder of the central nervous system, but it is unclear if this is linked to ER-phagy [108].

CCPG1 has a clear role in ER proteostasis *in vivo*. Genetrap mice that have an approximately 100-fold reduction in *Ccpg1* mRNA in the pancreas display a profound deficiency in proteostasis within pancreatic acinar cells [30]. These exocrine cells ordinarily contain an extensive rough ER producing large amounts of secretory enzymes. In the absence of CCPG1 and ER-phagy, the ER lumen becomes swollen with insoluble aggregates of enzymes and chaperones. This is visible ultrastructurally by transmission electron microscopy. It is unclear what the molecular mechanism is that links CCPG1-mediated ER-phagy to proteostasis. It is tempting to speculate that, as occurs indirectly with FAM134B, CCPG1 binds lumenal protein (directly or indirectly, via its lumenal domain or via interactions with other membrane-embedded intermediaries). Nonetheless, other primary deficiencies in ER function, such as block of secretion, lead to similar phenotypes as *Ccpg1* loss-of-function in pancreatic acinar cells [109], [110]. Notably, CCPG1-mediated proteostasis might occur in other “professional” secretory cells; gastric chief cells display a similar aberrant pathology to pancreatic acinar cells in histological sections from *Ccpg1* genetrap mice [30].

Another emergent function for ER-phagy is in responses to infection. FAM134B is required for resistance to infection of MEFs and endothelial cells with Ebolavirus and Flavivirus, respectively, although mechanistic information on how this occurs is lacking [111], [112]. FAM134B is cleaved by a Flavivirus-encoded protease within its RHD, ablating ER-phagy and leading to evasion of host virus restriction [112]. Conversely, RTN3-mediated membrane remodeling promotes Flavivirus proliferation, although whether this is related to ER-phagy and RTN3L isoform function, specifically, has not been tested [113]. Notably, upon infection with cells with living bacteria, a UPR response is engaged that appears to promote ER-phagy, albeit via an unknown receptor (not FAM134B, which was the sole candidate tested in this study) [114]. This ER-phagy appears to be required for immune signaling in response to the pathogen; the data are consistent with a model where early autophagy structures provide a signaling platform for the TBK1 (TANK-binding kinase 1) serine–threonine kinase, which is important in innate immune responses. The involvement of ER-phagy here could be related to the steady state localization of the upstream activator of TBK1, STING (stimulator of interferon genes), to the ER in unperturbed cells. Perhaps nascent autophagosomes bring together STING, TBK1 and potentially other factors required for signaling, although this requires deeper investigation, including identification of the ER-phagy receptor involved.

Finally, SEC62 is expressed at high levels in a number of carcinomas due to gene amplification. This positively correlates with progression [115], [116]. SEC62 amplification may be consistent with a double-edged sword role for the UPR in cancer progression; the UPR can drive cancer progression but paradoxically also engages cancer cell death [117]. Perhaps the overexpression of SEC62 (particularly non-stoichiometrically with SEC63 and SEC61 complexes) results in elevated recovER-phagy and toleration of high-level UPR signaling. Similarly, the sensitization of CCPG1-deficient pancreas to inflammation in aging mice suggests a potential role in pancreatic cancer, given the key role of inflammatory responses in genesis of this disease [30]. FAM134B is lost in colorectal cancer and may decrease cancer cell fitness *in vitro*. These observations point toward a tumor suppressor role for FAM134B-mediated ER-phagy, suggesting that targeting this pathway would not be a sensible approach in cancer [118], [119]. However, other reports show that in *IDH1*-mutant glioma, targeting FAM134B could kill tumor cells [120]. These observations are not necessarily paradoxical. For example, FAM134B loss-of-function might indeed promote tumorigenesis in those cell types where the consequent proteostatic defect was tolerable. However, in cell types already predisposed to stress from misfolded proteins, via mutation of another pathway such as *IDH1*, loss of the same process of ER-phagy might not be tolerable. FAM134B may thus constitute a good target for synthetic lethal therapeutic approaches in selected tumor genotypes. Alternatively, FAM134B-mediated ER-phagy may have fundamentally different mechanistic roles in different cancer types. Overall, the role of ER-phagy in cancer requires urgent investigation.

## Relation of ER-Phagy to Other Selective ER-to-Lysosome Degradation Pathways

The discussion of ER-phagy herein has focused on macroER-phagy. However, the following section briefly outlines some potentially macroautophagy-related pathways that have been described for selective transport of ER, or aberrant protein products from the ER, to the lysosome. A more comprehensive overview of these and their relationship to macroER-phagy can be found in another recent review [121].

In both yeast and mammals, selective microautophagy pathways act on the ER. These constitute bona fide examples of ER-phagy within the broad definition of this term. However, in contrast to macroautophagy, these pathways act by direct lysosomal (or vacuolar in yeast) engulfment of fragments of ER. In yeast, this is seen when ER stressors such as DTT cause the ER to expel a large, multi-layered “whorled” fragment of ER to counterbalance the expansion of the ER that the UPR engages [23], [25]. In mammals, budding of the ER from ER exit sites, which ordinarily operate in coatomer-protein dependent anterograde transport to the Golgi, and capture of these buds by lysosomes, participates in PC clearance [24]. It is unclear to what extent the molecular basis of this process overlaps with the previously mentioned clearance of PC by FAM134B-dependent macroautophagy of the ER [26].

The label ER-to-lysosome associated degradation (ERLAD) has recently been proposed as an umbrella term for all pathways that mediate degradation of aberrant protein from the ER by non-proteasomal, lysosomal routes (i.e., in opposition to ERAD) [26], [122]. Non ER-phagy mechanisms of ERLAD can thus complement ER-phagy in regulation of ER proteostasis. A notable, recently described example of non ER-phagy ERLAD shares some molecular players with ER-phagy. In this, FAM134B mediates clearance of an ERAD-resistant mutant of alpha-1-anti-trypsin (ATZ) [122]. However, this is distinct from ER-phagy-mediated clearance of PC at several levels. ATZ fills single, rather than double, membraned vesicles and the delimiting membrane is generated by budding of a vesicle from the ER, rather than a true phagophore, and this occurs independently of the ULK complex. However, the ERLAD vesicle does incorporate FAM134B and calnexin. Furthermore, it is decorated with lipidated GABARAP/LC3, and, as in macroautophagy, an STX17 and VAMP8 SNARE pairing mediates lysosomal fusion. However, the FAM134B–GABARAP/LC3 interaction plays no role in vesicle generation, instead facilitating this lysosomal fusion of the vesicle. Notably, there are also several other mutant protein species that are selectively lysosomally removed from the ER, and thus constitute potential examples of ERLAD. These include mutant dysferlin [36], granules of thyrotrophic hormone beta subunit (TSH-β) [123] and mutants of gonadotrophin-releasing hormone receptor (GnRHR) [124]. However, the degree of mechanistic overlap with ER-phagy in these instances is unknown. For example, TSH-β is found in ER-derived vesicles that acquire lysosomal markers, perhaps paralleling FAM134B-driven ERLAD of ATZ. Mutant GnRHR is found at the periphery of bona fide autophagosomes by ultrastructural studies, inconsistent with targeting by ER-phagy. However, as this is ordinarily a transmembrane protein, this could potentially be consistent with transfer to delimiting autophagic membranes derived from the ER during autophagosome formation [124]. However, overall, these pathways require further identification of mechanistic players to clarify or exclude any parallels with canonical ER-phagy.

## Outstanding Mechanistic and Functional Questions

This section highlights outstanding questions on the mechanism and functional importance of ER-phagy. The author urges the interested reader to investigate complementary perspectives in other recent reviews, such as those providing a comparison of ER-phagy with other proteostatic mechanisms regulating ER stress, or expanding philosophically on the justifications for more research into this fascinating process [83], [125].

### Signaling and recognition events in ER-phagy

Is cytosolic poly-ubiquitination of ER proteins important in ER-phagy, for example, via recruitment of ubiquitin-binding proteins that could contribute to recognition by the phagophore? Such ubiquitin-binding proteins might be cargo receptors shared in common with other selective autophagy pathways. The recruitment of the ubiquitin-binding cargo receptor p62/SQSTM1 to fragments of ER undergoing autophagy in liver could be an example of this, although this phenomenon requires further characterisation [93]. Similarly, a feature of some other selective autophagy pathways is the activation of TBK1, which phosphorylates receptors within extended LIR motifs and ubiquitin-binding domains, in order to increase their GABARAP/LC3 or ubiquitin affinity, respectively [70], [126]. AIM phosphorylation can also occur in yeast receptors [127]. The involvement of TBK1 activity or LIR phosphorylation in ER-phagy pathways is unknown, although CCPG1, in particular, contains a potentially phosphoregulable extended LIR (6-**S**D**S**D**SS**CGWTVISH; potentially phosphorylatable residues in bold, LIR motif positions 1 and 4 underlined). FIR or Atg11BR motif function in ER-phagy, for example, in CCPG1 and yeast Atg39, respectively, might also be regulable by phosphorylation, as suggested to occur for the FIR of p62/SQSTM1 [74] and for the Atg11BR of Atg32 [128].

We also need to know what events are sensed in order to stimulate ER-phagy from a particular locale within the ER. For example, could the removal of misfolded PC from within the ER involve lessened mobility of PC-bound chaperones such as calnexin, and consequent FAM134B clustering? Such hypothetical receptor clustering could be envisaged to play a role in stimulation of ER-phagy. For instance, it might contribute to local ER deformation or provide a platform to further stimulate phagophore growth (see below, “Function and interplay of ATG protein interactions”). Interestingly, GABARAP/LC3 localization to the ER precedes TEX264 recruitment upon nutrient-starvation [90]. This observation raises the possibility that, in at least some ER-phagy paradigms, signaling mechanisms also operate prior to early receptor clustering, in order to establish the initial phagophore. This is not mutually exclusive with determination of ER-phagy sites by receptor activity; superimposed upon upregulation of phagophore generation (either generally or at specific regions within the ER), localized activation of receptors would increase the probability of capture and consolidation of the ER-phagy response at phagophores within their vicinity.

Varying degrees of specificity in terms of sub-ER content turnover might be associated with different receptors. For example, with a potent, pan-cellular stimulus such as nutrient-starvation, it is possible that there is less localized control over ER-phagy initiation than with, for example, formation of a discrete aggregate of misfolded collagen in the ER lumen. FAM134B, at least, can operate in both paradigms. It would be interesting to see if TEX264, which makes perhaps the most significant contribution to nutrient-starvation induced ER-phagy turnover, had a similarly significant role in targeted proteostasis of a species such as misfolded collagen. It would also be highly informative to compare the impact of different receptors, downstream of different initiating stimuli, not just nutrient starvation, on the ER proteome. For instance, CCPG1 is incorporated into ER autophagosomes that are distinct from those labelled by FAM134B and TEX264 [90], and CCPG1 is partially redundant with FAM134B and TEX264 for ER-phagic flux under nutrient starvation [91]. Do CCPG1 and/or FAM134B account for the turnover of a portion of the nutrient-starvation sensitive ER proteome that is relatively less dependent on TEX264 (but still sensitive to core autophagy inhibition)?

Where there is evidence of selective protein turnover during ER-phagy, might ER-phagy receptors other than FAM134B, such as RTN3L, SEC62, CCPG1 and TEX264, recognize lumenal protein via intermolecular interactions (Fig. 4)? This is not mutually exclusive with specificity imposed via localization to specific subdomains of the ER. In a recognition model, CCPG1 could potentially bind to lumenal cargo, or adaptors for cargo, via its lumenal domain. TEX264 also has lumenal N-terminal region, although this is extremely short. All four receptors could bind other membrane-embedded chaperones or adaptors, as per FAM134B. Alternatively, sensing of locally concentrated lumenal cargo could be transduced by as-yet-unknown mechanisms driving recruitment of receptors without any direct or indirect interaction with the cargo protein (Fig. 4). Other than RTN3L–RTN3L, FAM134B–ATL2, and potentially, ATL3–ATL3, homotypic and heterotypic interactions between cargo receptors or ancillary proteins involved in membrane reshaping have not been extensively explored. It is possible that binding of cargo receptors with intrinsic reshaping potential to receptors that link to specific lumenal cargo could co-ordinate both of these principles of ER-phagy (Fig. 3, Fig. 4). Notably, formation of such homo- and heterotypic interactions could represent a key signal transduction regulable step in ER-phagy. In this vein, FAM134B and TEX264 are simultaneously recruited to active ER-phagy sites. TEX264 does not have intrinsic reshaping activity, and it is plausible that this is contributed by FAM134B. On the other hand, the targeting of FAM134B and TEX264 into growing ER autophagosomes appears mutually independent [90], and FAM134B and TEX264 are partially redundant in promoting ER-phagy flux [91]. Nonetheless, the regulated interaction, or at least functional co-operation, of different receptors at particular ER sites, or downstream of particular ER-phagy stimuli, could provide exquisite control of different forms of ER-phagy.

Finally, it is also likely that not all ER-phagy pathways are primarily involved in proteostasis. For instance, ER-phagy might participate in targeted degradation of ER with aberrant lipid content or with topological abnormalities. It is also possible that individual receptors could flexibly bind a range of different cargoes or adaptors to participate in different forms of proteostasis or different alternate functions of ER-phagy, depending on the prevailing cell state and signaling conditions. Indeed, the full scope of individual receptor function in regulation of ER physiology remains to be determined.

### Function and interplay of ATG protein interactions

Why do some ER-phagy receptors have multiple ATG-protein interacting motifs? For example, RTN3L and ATL3 each have more than one LIR motif [88], [92]. This multivalency for ATG proteins is further accentuated in molecules that can multimerize, such as RTN3L. It is possible that initial recruitment of receptors by occupancy of some GABARAP/LC3 binding sites operates a positive feedback loop, resulting in further local recruitment of GABARAP/LC3 and consolidation of the receptor binding. This could be important in imparting irreversibility on the ER-phagy process once initiated. A specific interesting example among mammalian cargo receptors is CCPG1, which binds to two different ATG protein species, GABARAP/LC3 and FIP200 (FAK-interacting protein 200 kDa). In other forms of selective autophagy, it is emerging that receptors may be recruited to cargo at the future site of phagophore generation and play a role in recruitment of the machinery that drives this process, rather than merely having a passive function in linking the cargo to the phagophore. For example, NDP52 may recruit the ULK complex to bacteria or mitochondria via FIP200 interaction [73], [75]. Could CCPG1 recruit initiating ATG proteins via FIP200 interaction and then subsequently tether to GABARAP/LC3 (Fig. 2)? Indeed, endogenous CCPG1 binds ULK1, presumably through its direct interaction with FIP200, although no evidence has been published for endogenous binding of ATG13 or ATG101 [30]. In an alternative example, p62/SQSTM1 multimers were shown to bind FIP200 and GABARAP/LC3 mutually exclusively, first FIP200 on the phagophore and then GABARAP/LC3 on the nascent autophagosome, imparting directionality on the autophagy process and ensuring retention of cargo [74]. However, it should be noted that, unlike p62/SQSTM1, there is no evidence that CCPG1 binding to GABARAP/LC3 and FIP200 is mutually exclusive.

### Physiological functions of ER-phagy revealed by receptor knockout

It is unlikely that we have uncovered the entire cohort of ER-phagy receptors; the tissue-specific effects, or lack of effects, of *in vivo* knockout or genetrap of *Fam134b*, *Rtn3* and *Ccpg1* suggest this [30], [85], [107]. It is an attractive proposition that different ER-phagy pathways exist in order to regulate different aspects of ER biology, which vary widely between different subregions of ER within the same cell type, and between different specialized cell types. Indeed, analysis of a panel of tissues from wild-type and autophagy-deficient mice showed that, in contrast to TEX264, which is ubiquitously expressed and subject to basal autophagic degradation, CCPG1 undergoes prominent autophagic turnover mostly in the pancreas and stomach [91], consistent with the tissue-restricted effects of its knockout. These potentially specialized effects of ER-phagy receptors should be taken into account when using data obtained from nutrient starvation in cultured cells to support the primacy of particular receptors in ER-phagy. Different physiological functions of ER-phagy will likely depend on individual receptors to differing degrees. Alternatively, there could be “core” ER-phagy receptors that function ubiquitously in ER-phagy pathways in conjunction with various different partner receptors, dependent on context. There are insufficient data yet to conclude which hypothesis is correct.

It is also possible that ER-phagy is not an essential physiological process in all unstressed cell types, and that challenges such as aging, exposure to infectious agents or mutation of cancer proto-oncogenes might be required to reveal the complete set of roles for ER-phagy receptors. Indeed, ER-phagy appears disrupted in a mouse model of progeria wherein overexpressed *Slc33a1* leads to accelerated aging, although whether the loss of ER regulation contributes to the aging phenotype is not clear [129]. It is also worth considering that published data have tended to describe the effect of individual targeting of receptors in cultured cells or *in vivo.* Investigating redundancy between ER-phagy pathways is not a trivial experimental challenge, but could yield insight into the broader relevance of these pathways in physiology and better define mechanistic overlaps. It is also important to address whether all isoforms of a receptor protein participate in ER-phagy. For example, only RTN3L, among RTN3 isoforms, has LIR motifs and thus stimulates ER fragmentation and ER-phagy [88]. Equally, CCPG1 has multiple isoforms that all have N-terminal ATG-binding regions but differ substantially in distal polypeptide sequence. A role in ER-phagy has only been explored for the canonical 757-amino-acid isoform (Table 1). Thus, isoform-specific knockouts may be required to reveal ER-phagy functions, especially if targeting of non-participating isoforms has confounding effects. Conversely, it should be noted that knockout of any given cargo receptor may have effects on pathways other than ER-phagy. For instance, a mechanistically distinct role for FAM134B in ATZ proteostasis by ERLAD is known [122]. As research progresses into the cellular functions of the other receptors, it is highly possible that ER-phagy-independent roles in ER regulation will be uncovered. In the long term, therefore, extra information will be required to ascribe the phenotypic effects of any cargo receptor knockout to ER-phagy per se.

### Potential other roles for ER-phagy

Historically, several processes have been observed at the ultrastructural level to correlate with degradation of ER fragments by macroautophagy, for example, the recovery of hepatic cells after phenobarbital treatment [130], or the clearance of intralumenal protein inclusions from the acinar cells of guinea pig pancreata after cobalt exposure [109],[110]. These should be tested for involvement of ER-phagy. For instance, the accumulation of ER lumenal inclusions in pancreata seems highly likely to be a CCPG1-regulated process [30].

Conversely, a number of physiological aberrancies that have been observed after loss of core autophagy protein function correlate with ER dysregulation. One explanation is that loss of general autophagy, or selective autophagy pathways other than ER-phagy, might indirectly impact on the ER. However, ER-phagy deficiency per se may directly underpin such phenotypes. Situations where the potential role of ER-phagy should be tested include in mouse chondrocytes, where *Atg7* is required to prevent PC accumulating within the lumen of the ER, raising the possibility that ER-phagy is critical for bone growth and homeostasis [131]. Similarly, *Atg5* is required in T cells to limit the volume of the ER. Defective calcium signaling is also seen in *Atg5*-deficient T cells [132]. This suggests that ER-phagy might influence lymphocyte calcium signaling and immune function. Again, discovery of an ER-phagy receptor implicated in this process would give the hypothesis credence. Finally, *Atg5* is required for restraint of ER expansion and immunoglobulin (Ig) synthesis in plasma cells (plasma cells are Ig-secreting cells formed after activation of B cells during infection). Here, autophagy appears to balance beneficial Ig synthesis against the elevated ER stress and consequent UPR-associated plasma cell death linked to overproduction of Ig [133]. Interestingly, CCPG1 expression is markedly increased during formation of plasma cells, so it is a strong candidate receptor to test here [134].

## Perspective: Translational Application of ER-Phagy Knowledge

This review has outlined mechanisms of ER-phagy and its role in disease. In addition to addressing shortcomings in our understanding of these, the other major challenge for the future is to consider translational aspects of the knowledge generated. Specifically, understanding of ER-phagy mechanism and function across the vista of potential health and disease settings, including identification of all involved molecules, will stimulate efforts for therapeutic intervention. Which molecules within the ER-phagy pathway might constitute therapeutically beneficial, druggable targets? In the majority of scenarios described thus far, upregulation of ER-phagy would be desirable. It may be possible to develop agents to activate scaffold molecules, such as ER-phagy receptors. However, this may prove difficult and the relative ease of identifying enzymatic activities for drug targeting underscores the need to identify signaling pathways negatively regulating ER-phagy. In addition, where ER-phagy networks are known to be directly suppressed by mutation or downregulation of a core component, for example, as occurs with FAM134B in HSAN type II, it could be useful to target residual ER-phagy activities. For instance, could strategies be developed to activate the expression or activity of redundant receptors, ordinarily active at low levels in the relevant cell type, to compensate for loss-of-function of the main pathway? In some instances, it may be that discrete cellular machineries such as ERAD or ERLAD, or microER-phagy, could be upregulated to compensate. Also, in the era of personalized medicine, genome editing techniques, for example, those based on CRISPR/Cas9 technology, might allow correction of loss-of-function mutations in ER-phagy proteins in sufficient numbers of cells, or in stem cells, in order to ameliorate the disease phenotype. Finally, in other scenarios, inhibition of ER-phagy may have some benefit. For example, if SEC62 amplification in cancer does permit aberrant cancer cell survival, inhibition of this pathway could be envisaged as a therapeutic option.

## Conclusion

In analogy to other forms of selective autophagy, such as mitophagy and xenophagy, efforts to uncover the fundamental mechanistic principles and functions of the emergent process of selective ER-phagy will benefit human health.
